# Supplementary material for: Altering the redox status of Chlamydia trachomatis directly impacts its developmental cycle progression
Source: eLife. 2025 Jan 17;13:RP98409. doi: 10.7554/eLife.98409 (PMC11741522; doi:10.7554/eLife.98409)
Supplement: Supplementary file 2. [file elife-98409-supp2.docx]

**Supplementary Table S1. List of Plasmids, Strains, and Primers**

| **Construct Plasmid** | **Relevant genotype** | **Ori** | **Source of Reference** |
| --- | --- | --- | --- |
| pBOMBL12CRia::L2 | *bla* P_Nm_::*gfp* P_tet_::As_dCas12vaa | pUC19 | (Ouellette et al., 2021) |
| pBOMBL12CRia(NT)::L2 | *bla* P_Nm_::*gfp* P_tet_::As_dCas12vaa  P*_dnaKmut_*::As_crRNA_NT | pUC19 | (Reuter et al., 2023) |
| pBOMBL12CRia(*ahpC*)::L2 | *bla* P_Nm_::*gfp* P_tet_::As_dCas12vaa  P*_dnaKmut_*::As_crRNA_*ahpC* | pUC19 | This study |
| pBOMBL12CRia(*ahpC)*- *ahpC*::L2 | *bla* P_Nm_::*gfp* P_tet_::As_dCas12vaa- *ahpC*  P*_dnaKmut_*::As_crRNA_*ahpC* | pUC19 | This study |
| pBOMB-G::L2 | *bla* P*_tet_*::mCherry | pUC19 | (Wood et al., 2020) |
| pBOMBDC::L2 | *bla* P*_tet_*::mCherry P_hctB__mKate P_euo__nGreen | pUC19 | This study |
| pBOMBDC-*ahpC*::L2 | *bla* P*_tet_*::*ahpC* P_hctB__mKate P_euo__nGreen | pUC19 | This study |
| pBOMBL12CRia::L2 (spec) | *aadA* P_Nm_::*gfp* P_tet_::As_dCas12vaa | pUC19 | (Shen et al., 2024) |
| pBOMBL12CRia(*ahpC*)::L2 (spec) | *aadA* P_Nm_::*gfp* P_tet_::As_dCas12vaa  P*_dnaKmut_*::As_crRNA_*ahpC* | pUC19 | This study |

| ***E. coli* Strain** | **Relevant genotype** | **Source of Reference** |
| --- | --- | --- |
| NEB10-beta | *Δ(ara-leu) 7697 araD139 fhuA ΔlacX74 galK16 galE15 ϕ80dlacZΔM15 (e14-) recA1 relA1 endA1 nupG rpsL (*Str^R^*) rph spoT1 Δ(mrr-hsdRMS-mcrBC)* | New England BioLabs |

| **Primer name** | **Sequence** | **Features** | **Usage** |
| --- | --- | --- | --- |
| PhctB_mK/(pB-G)/5’LIC | ctggccttttgctcacatggaattgTGTTAAAAACTAACCATTTTTTATTAAAG | Insert P_hctB__mKate into pBOMB-G; overlaps P_euo__nGreen | Generate pBOMBDC::L2 |
| PhctB_mK/(Peuo)/3’LIC | tttgttaaaaataCGTCTTAGGAGCTTTTTG | Insert P_hctB__mKate into pBOMB-G; overlaps P_euo__nGreen | Generate pBOMBDC::L2 |
| Peuo_nG/(mK)/5’LIC | agctcctaagacgTATTTTTAACAAACCACTTGATTAATAAG | Insert P_euo__nGreen into pBOMB-G; overlaps P_hctB__mKate | Generate pBOMBDC::L2 |
| Peuo_nG/(pB-G)3’LIC | ttgccagaaaaaacacctttaggcgCTAAGCTACTAAAGCGTAGTTTTC | Insert P_euo__nGreen into pBOMB-G; overlaps P_hctB__mKate | Generate pBOMBDC::L2 |
| *ahpC*/(pBOMB)/5'LIC | gatctaaagaggagaaaggatctgcATGCCAGCTATGCGCCTTATTG | lower case for plasmid overlap construction | for amplification of *ahpC* into pBOMBDC |
| *ahpC*/(pBOMBDC)3’LIC | acatatttgaatggtcgaccggtacTTAATCCATCGTCTGGAAG | lower case for plasmid overlap construction | for amplification of *ahpC* into pBOMBDC |
| *ahpC*/(pBOMBL12CRia)/F | cgcaacgtagctgcttaagtaccggaggaatctgcATGGGATCATTAGTTGGAAG | lower case for plasmid overlap construction | For insertion of *ahpC* 3’ of *dCas12* in pL12CRia |
| *ahpC*(pBOMBL12CRia)/R | catgagcggatacatatttgaatggTTAATCCATCGTCTGGAAG | lower case for plasmid overlap construction | For insertion of *ahpC* 3’ of *dCas12* in pL12CRia |
| ct603 *ahpC* | TCCGTTGACGACATTGAGAC | Forward qPCR primer | For qPCR of *ahpC* |
| ct603 *ahpC* | AGGGTCTGCTAACAGAGGATA | Reverse qPCR primer | For qPCR of *ahpC* |
| ct443 *omcB* | CGGTAGGATCTCCCTATCCTATT | Forward qPCR primer | For qPCR of *omcB* |
| ct443 *omcB* | CGAACTCTGCTTCACATGGTA | Reverse qPCR primer | For qPCR of *omcB* |
| ct743 *hctA* | AAGCTAAAGCTGCTGCTAAGA | Forward qPCR primer | For qPCR of *hctA* |
| ct743 *hctA* | GTTGGTTTGACCTTTGCTTTAGT | Reverse qPCR primer | For qPCR of *hctA* |
| ct046 *hctB* | AACTGTAGCAGCTCGTAAGC | Forward qPCR primer | For qPCR of *hctB* |
| ct046 *hctB* | TTTGCGAGCTACAGTCTTCTT | Reverse qPCR primer | For qPCR of *hctB* |
| ct798 *glgA* | GAGCAGAAAGGTCCTCACTTTA | Forward qPCR primer | For qPCR of *glgA* |
| ct798 *glgA* | TCCCGTAGCAGGTACCTATAAT | Reverse qPCR primer | For qPCR of *glgA* |
| ct441 *tsp* | GTAGCCTTCGTGTAGGTGATATT | Forward qPCR primer | For qPCR of *tsp* |
| ct441 *tsp* | AGGATCCTGGAGAACCTCTT | Reverse qPCR primer | For qPCR of *tsp* |

| **gBlock Name** | **Sequence** | **Features** | **Usage** |
| --- | --- | --- | --- |
| *ahpC_*IGR crRNA | tgtgaaagtgggtcttaagacgtcggtactgcatgtgacgcacgtagatcatgca*TTCACCGGTGGAGACGGTTTTCTTATAATGACACC*TAATTTCTACTCTTGTAGAT**TAGGAGGAGTACTATGGGATC**CAAATAAAACGAAAGGCTCAGTCGAAAGACTGGGCCTTTCGTTTTATcaacagcggtctactgaatctgagctagtgcgtgatataattaaaattatattca | Lower case for plasmid overlap and spacer, *italicized* for P_dnaKmut_ promoter sequence, underlined for crRNA scaffold, **bold** for *ahpC*_IGR targeting sequence, Upper case for rrnB1 terminator | Insert into BamHI-digested pBOMBL12CRia::L2 plasmid |

Ouellette, S. P., Blay, E. A., Hatch, N. D., & Fisher-Marvin, L. A. (2021). CRISPR Interference To Inducibly Repress Gene Expression in Chlamydia trachomatis. *Infect Immun*, *89*(7), e0010821. <https://doi.org/10.1128/IAI.00108-21>

Reuter, J., Otten, C., Jacquier, N., Lee, J., Mengin-Lecreulx, D., Lockener, I., Kluj, R., Mayer, C., Corona, F., Dannenberg, J., Aeby, S., Buhl, H., Greub, G., Vollmer, W., Ouellette, S. P., Schneider, T., & Henrichfreise, B. (2023). An NlpC/P60 protein catalyzes a key step in peptidoglycan recycling at the intersection of energy recovery, cell division and immune evasion in the intracellular pathogen Chlamydia trachomatis. *PLoS Pathog*, *19*(2), e1011047. <https://doi.org/10.1371/journal.ppat.1011047>

Shen, L., Gao, L., Swoboda, A. R., & Ouellette, S. P. (2024). Targeted repression of topA by CRISPRi reveals a critical function for balanced DNA topoisomerase I activity in the Chlamydia trachomatis developmental cycle. *mBio, 15(2)*, e0258423. <https://doi.org/10.1101/2023.03.14.532001>

Wood, N. A., Blocker, A. M., Seleem, M. A., Conda-Sheridan, M., Fisher, D. J., & Ouellette, S. P. (2020). The ClpX and ClpP2 Orthologs of Chlamydia trachomatis Perform Discrete and Essential Functions in Organism Growth and Development. *mBio*, *11*(5). <https://doi.org/10.1128/mBio.02016-20>
